# Effects of fundamental movement skills on health-related quality of life in Chinese school-age children: the mediating role of physical fitness level

**DOI:** 10.3389/fpubh.2023.1023662

**Published:** 2023-05-03

**Authors:** Shuqing Xie, Yulan Zhou, Yanmin Yin, Rui Shao, Lei Fang, Weide Shao

**Affiliations:** College of Physical Education and Health Sciences, Zhejiang Normal University, Jinhua, Zhejiang, China

**Keywords:** school-age children, fundamental movement skills, health-related quality of life, physical fitness level, mediating effect

## Abstract

**Background:**

The primary purpose of this study is to analyze the relationship between school-age children’s fundamental movement skills (FMS), physical fitness levels, and the health-related quality of life (HRQoL); To explore the mediating role of physical fitness levels between school-age children’s FMS and HRQoL.

**Methods:**

In the cross-sectional survey in 2021, 334 school-age children aged 6–10 (8.20 ± 1.16) were recruited from primary schools in Jinhua City, Zhejiang Province, China. Test of Gross Motor Development 2 (TGMD-2), National Standards for Students’ Physical Health, and Pediatric Quality of Life Inventory TM Version 4.0 (PedsQL™ 4.0) were used to investigate the FMS, physical fitness level, and HRQoL of school-age children. Hierarchical regression was used to analyze the relationship among FMS, physical fitness levels, and HRQoL. Bootstrap is used to evaluate the mediating role of physical fitness levels in the relationship between FMS and HRQoL.

**Results:**

The higher the FMS and physical fitness, the higher the school-age children’s HRQoL, physical functioning, social functioning, and school functioning (*r* = 0.244–0.301, *p* < 0.01). In addition, developing children’s FMS promotes physical fitness levels (*r* = 0.358, *p* < 0.01). The regression analysis results of controlling gender, age, and body mass index z (BMI-z) scores showed that FMS significantly positively predicted the physical functioning (*β* = 0.319, *p* < 0.01), social functioning (*β* = 0.425, *p* < 0.01), and school functioning (*β* = 0.333, *p* < 0.01) of school-age children. When the physical fitness level enters the regression equation, the absolute value of the regression coefficient of FMS decreases. However, it can still significantly predict the physical functioning (*β* = 0.211, *p* < 0.01) and school functioning (*β* = 0.142, *p* < 0.05) of school-age children. Simple intermediary analysis shows that physical fitness level plays an intermediary role between FMS, physical functioning (indirect effect = 0.089 [95% Confidence interval (CI) = 0.015,0.195]), and school functioning (indirect effect = 0.065 [95% CI = 0.007,0.150]).

**Conclusion:**

This study shows that physical fitness levels mediate the relationship between FMS and HRQoL. Encouraging the development of FMS and promoting physical fitness levels of school-age children can effectively improve the HRQoL of school-age children.

## 1. Introduction

Health-related quality of life (HRQoL) is an individual’s subjective evaluation of his physical functioning, mental state, and social ability, which involves many aspects such as physiology, psychology, and social communication (1). HRQoL can effectively evaluate an individual’s health level comprehensively. School-age children aged 6–10 are in a critical period of physical and mental development and social development. The level of HRQoL in this period can affect the level and quality of their adult life (2). Compared with adults, school-age children’s HRQoL has long been neglected (3). The World Health Organization has recently pointed out that children’s health and well-being are crucial to society’s health and sustainable development (4). School-age children’s HRQoL has attracted more and more attention (5). In order to better evaluate children’s HRQoL, researchers have developed different measuring tools, such as the Child Health Questionnaire (CHQ) Questionnaire for Measuring Health-Related Quality of Life in Children and Adolescents (KINDLR), Pediatric Quality of Life Inventory 4.0 (PedsQL™4.0), etc. (6–8). PedsQL™4.0 is mainly used to measure the HRQoL of children and adolescents aged 2–18, including 23 items in four parts: physical functioning, school functioning, social functioning, and emotional functioning (6). The scale has been translated into different languages and is widely used in different cultures, and it is a mature tool to evaluate children’s HRQoL at present (9).

Fundamental movement skills (FMS) are the ability to coordinate basic motor skills. It is the foundation for people to carry out complex physical and sports activities, including locomotion, object control, and stability skills (10). FMS is also a fundamental element of the overall development of school-age children. It is used in almost every aspect of individual daily activities, such as running, catching, throwing, writing, bathing, dressing, etc.; it plays a vital role in children’s physical, social, and mental health (11). More and more studies have explored the relationship between children’s FMS development and various health-related outcomes, including social, cognitive, and emotional development (12). Piek et al. (13) and Herrmann et al. (14) found that children with high FMS levels are more popular than those with poor FMS and better integrate into peer groups. In contrast, a low FMS level will lead to negative interpersonal relationships (problems with peers) and internal (low self-evaluation) consequences at the psychosocial level, which will impact mental health. In addition, studies have shown that better FMS in school-age children is related to higher cognitive ability and better academic performance (15). However, most of the research on the overall level of FMS and HRQoL focuses on school-age children with dyskinesia or diseases (16). Only Raz-Silbiger et al. (17) and Redondo-Tebar et al. (16) discussed the relationship between FMS and HRQoL in typical developmental children. Raz-Silbiger et al. (17) found a positive correlation between object control skills and HRQoL in studying the relationship between children’s FMS and HRQoL. Among them, the children’s object control score is positively correlated with the physical and social functioning of HRQoL. The research of Redondo-Tebar et al. (16) shows that a good FMS development level is related to a good HRQoL, mainly for children with high FMS levels, whose scores in physical functioning, emotional functioning, social functioning, and school functioning are significantly higher. Therefore, the development of FMS can promote the HRQoL level of school-age children. At present, however, there are few empirical studies on the relationship between FMS and HRQoL in typical developmental school-age children, and more studies are needed to explore the relationship between FMS and HRQoL further.

As we all know, physical fitness levels have many benefits for school-age children’s physical health (i.e., cardiovascular and metabolic diseases, obesity, and musculoskeletal problems) and mental health (i.e., depression, anxiety, stress, and quality of life) (18–21). As an essential health indicator in the development of school-age children, physical fitness levels can be regarded as a comprehensive measurement of physical functioning, including cardiopulmonary endurance, muscle strength, endurance, body composition, and flexibility (22). More and more studies have confirmed that FMS plays a crucial role in the physical fitness levels of school-age children (23). Barnett et al. (24) studied the relationship between FMS and physical fitness in 1045 school-age children aged 7.9–11.9. The research showed that school-age children with poor FMS development also had poor physical fitness levels. At the same time, they also found that FMS in childhood, especially object control skills, can significantly predict physical fitness levels in adolescence. Furthermore, physical fitness levels can affect children’s HRQoL. Studies have shown that school-age children with high physical fitness levels score higher than school-age children with low physical fitness levels in physical and mental health, social fields, academic performance, and overall HRQoL level (25, 26). Because of the above findings, we speculate that FMS may affect the physical fitness levels of school-age children, thus affecting HRQoL. However, no report exists on the relationship among FMS, physical fitness levels, and HRQoL in school-age children. This study aims to analyze the relationship between children’s FMS, physical fitness levels, and HRQoL; To explore the intermediary role of physical fitness levels between children’s FMS and HRQoL. Based on the literature review, we proposed the following hypotheses: (1) FMS positively correlates with HRQoL. (2) Physical fitness levels are positively correlated with HRQoL. (3) Physical fitness levels partially mediate between FMS and HRQoL in school-age children. The findings of this study will enrich the theoretical system between FMS, physical fitness levels, and HRQoL and provide new theoretical approaches to promote HRQoL levels in school-aged children.

## 2. Method

### 2.1. Participants

From October to mid-November 2021, FMS, physical fitness levels, and HRQoL of 334 healthy children aged 6–10 in public primary schools in Jinhua City, Zhejiang Province, China, were investigated. TGMD-2 and National Student Physical Health Standards (2014 Revision) are adopted to test school-age children’s FMS and physical fitness levels. Parents of school-age children complete the HRQoL questionnaire and return it to teachers within 1 week. A total of 330 questionnaires were distributed, and 304 questionnaires were collected, with an effective recovery rate of 92%. There are 150 boys (8.14 ± 1.22 years old) and 154 girls (8.27 ± 1.10 years old). The Ethics Committee of Zhejiang Normal University approved the survey, and the parents of 330 participants provided informed consent.

### 2.2. Instruments and procedures

#### 2.2.1. Fundamental movement skills

The study used the Test of Gross Motor Development-2nd edition (TGMD-2) to assess children’s FMS. The TGMD-2 includes two subscales: locomotor (e.g., running, galloping, leaping, horizontal jumping, sliding, and hopping) and object-control (e.g., striking, dribbling, rolling, throwing, catching, and kicking) skills (27). Each skill contains 3–5 scoring criteria. If the skill performance meets one criterion, one point will be scored, and if it does not, 0 points will be scored. The combination of all skills was summed to give a total FMS score, while locomotor and object-control subtest scores were created by totaling the skills scores within each subscale. The total score of the locomotor and object control skills test is 96, and the higher the score, the higher the FMS level of the children.

During the test, the evaluators will explain and demonstrate the skills and movements and give school-age children two practice opportunities. During the test, four school-age children were tested as a group. Children were tested twice for each skill movement, and the sum of the two scores was the final score for the school-age children’s FMS. The assessors videotaped the entire test process. At the end of the test, two assessors led by one expert independently scored the video. For skills the assessors were unsure of or disputed by the scoring, the skills performed by the school-age children were replayed and re-evaluated by the experts. According to the Pearson correlation coefficient method, the reliability among raters is *r* = 0.709 ~ 0.889, which indicates that raters have good consistency.

#### 2.2.2. Health-related quality of life

Pediatric Quality of Life Inventory TM Version 4.0 (PedsQL TM 4.0) is a tool developed by Children’s Hospital and Health Center in San Diego, California, USA, which is used to measure the HRQoL of children aged 2–18 years (6). It consists of a self-report and a parent-agent report. The self-report and parent-agent report items are essentially the same but differ in language expression. Children’s self-report scale is expressed in the first person, while the parent-agent scale is expressed in the third person. Each entry asks how often something happened in the last month. Studies have shown high consistency between parent-agent reports and self-report scales (28). In this study, young school-age children have problems expressing their feelings, understanding the items on the HRQoL tool, and reporting their own perceived HRQoL. Therefore, parents’ proxy report is adopted to measure the HRQoL level of school-age children. The scale includes four dimensions: physical functioning (8 items), emotional functioning (5 items), social functioning (5 items), and school functioning (5 items), with a total of 23 items. Each item is used to inquire about the frequency of something happening in the last month, and it is graded according to 5 levels: 0 = never; 1 = seldom; 2 = sometimes; 3 = almost always;4 = always. All entries have equal weights and are scored in reverse and then converted into a percentage system (0 = 100, 1 = 75, 2 = 50; 3 = 25; 4 = 0). The scores for each dimension and the total score for overall quality of life were the sums of the entries to which they belonged divided by the number of entries included, with higher scores indicating a better health-related quality of life. The reliability and validity of PedsQL ™ 4.0 are Cronbach’s *α* = 0.847 and KMO = 0.834, respectively, so the reliability and validity of this scale are good.

#### 2.2.3. Physical fitness level

The National Student Physical Health Standard (Revised in 2014) is China’s official measurement and evaluation standard of students’ physical fitness levels. Several experts in China conclude the test content after a lengthy investigation and revision, and it has strong applicability to children in China. Physical fitness levels for school-age children are adopted according to the corresponding items in the National Student Physical Health Standards (revised in 2014), mainly including body mass index (BMI), vital capacity, 50 m running, sitting body flexion, one-minute jump rope, and one-minute sit-ups (29). Height was measured with a portable stadiometer (Seca 213, Hamburg, Germany), accurate at 0.1 cm level. Body weight was measured using an electronic calibrated scale (Tanita TBF- 300A, Illinois, USA), accurate at 0.1 kg level. Participants were barefoot and dressed in light clothes. Body mass index, BMI) is calculated by dividing weight (kg) by the square of height (m), and BMI – z scores were calculated using the ‘LMS’ method (30). Considering the development characteristics of children’s physical and mental health at different ages, the physical health scores of different ages account for different items (the full score is 100). The total score of physical fitness levels is obtained by inputting all the data into excel and adding them according to specific weights (Table 1). The higher the score, the higher the physical fitness levels of children.

**Table 1:** Test items.

Age	Test item	Score
6–8 years	BMI	15
	Vital Capacity	15
	50 m running	20
	Sitting body flexion	30
	One-minute jump rope	20
8–10 years	BMI	15
	Vital capacity	15
	50 m running	20
	Sitting body flexion	20
	One-minute jump rope	20
	One-minute sit-ups	10

### 2.3. Data analysis

The statistical software SPSS26.0 was used to analyze the data, and the significance level was set as *p* < 0.05. Regular test (skewness and kurtosis) is carried out on the measured data to check the distribution of the data. As the data is normally distributed, the following statistical methods are adopted. Firstly, school-age children’s FMS, HRQoL, and physical fitness levels are statistically described and presented as mean and standard deviation. The gender difference was analyzed by independent sample *t*-test. Secondly, the Pearson correlation coefficient tests the relationship among FMS, physical fitness levels, and HRQoL. Correlation coefficient values of 0.1, 0.3, and 0.5 are considered small, medium, or large (31). Secondly, existing studies have shown that school-age children’s gender, age and BMI will affect their HRQoL (3, 4). Therefore, gender, age, and BMI *z* scores are included in the regression model to control (the gender setting is virtual, in which boys =0 and girls =1). Hierarchical linear regression explored the relationship between FMS, physical fitness levels, and HRQoL in school-age children. In the regression analysis, four dimensions of HRQoL are selected: physical functioning, social functioning, emotional functioning, and school functioning. Finally, the Process plug-in in SPSS compiled by Hayes was used to test the mediating effect. The percentile Bootstrap of deviation correction was used to evaluate the mediating effect of physical fitness levels between FMS and school-age children’s HRQoL (32). If the 95% confidence interval does not include 0, the mediation effect is significant, and the amount of mediation effect is the ratio between the indirect effect and total effect (33).

## 3. Results

### 3.1. Descriptive statistics of research variables

Table 2 shows the characteristics of the participants. Three hundred thirty-four school-age children participated in this test, and 30 did not complete FMS, physical fitness levels, and HRQoL questionnaire because of illness, leave, and other reasons. Finally, 304 participants completed all indicators (FMS, physical fitness levels, and HRQoL questionnaire). The BMI of the participants was 16.89 ± 2.63, and the BMI-z scores were 0.43 ± 1.15. The independent sample T-test results showed no statistical difference in age, height, weight, BMI, and BMI-z scores between boys and girls (*p* > 0.05). Boys scored higher in FMS and physical functioning than girls, and girls scored higher in physical health, emotional, and school functioning than boys (*p* < 0.05). Overall, the average score of FMS of school-age children is 65.87 ± 8.06. The average score of HRQoL was 80.22 ± 8.73, including physical functioning 83.87 ± 10.25, emotional functioning 76.79 ± 14.30, social functioning 83.14 ± 11.21 and school functioning 77.07 ± 11.14. The average score of physical fitness levels is 81.31 ± 8.31.

**Table 2:** Participant characteristics.

Index	Total sample (*N* = 304)	Boys (*N* = 150)	Girls (*N* = 154)	*p*
Age(yr)	8.20 ± 1.16	8.14 ± 1.22	8.27 ± 1.10	0.355
Height(cm)	129.97 ± 10.35	130.60 ± 9.08	129.35 ± 11.45	0.292
Weight(kg)	29.07 ± 7.50	29.79 ± 8.40	28.37 ± 6.45	0.100
BMI(kg/m^2^)	16.89 ± 2.63	17.17 ± 2.92	16.61 ± 2.28	0.064
BMI – z scores	0.43 ± 1.15	0.53 ± 1.23	0.34 ± 1.05	0.147
FMS	65.87 ± 8.06	67.17 ± 8.10	64.60 ± 7.85	0.005^**^
Physical functioning	83.87 ± 10.25	85.75 ± 9.71	82.04 ± 10.46	0.002^**^
Emotional functioning	76.79 ± 14.30	74.47 ± 14.19	79.06 ± 14.08	0.005^**^
Social functioning	83.14 ± 11.21	82.67 ± 11.44	83.60 ± 11.00	0.467
School functioning	77.07 ± 11.14	75.60 ± 11.40	78.51 ± 10.73	0.023^*^
HRQoL	80.22 ± 8.73	79.62 ± 8.60	80.80 ± 8.86	0.239
Physical fitness levels	81.31 ± 8.31	80.13 ± 8.57	82.46 ± 7.91	0.014^*^

*p < 0.05; **p < 0.01.

### 3.2. Correlation analysis of research variables

Table 3 shows the correlation analysis of school-age children’s FMS, HRQoL, and physical fitness levels. It can be seen from Table 3 that FMS of school-age children has a small to medium correlation with physical functioning, social functioning, school functioning, and HRQoL (*R* = 0.244–0.301, *p* < 0.01) but no significant correlation with emotional functioning (*R* = −0.029, *p* > 0.05). There is a weak correlation between physical fitness levels and physical functioning, social functioning, school functioning, and HRQoL (*r* = 0.221–0.263, *p* < 0.01) but no significant correlation with emotional functioning (*r* = 0.047, *p* > 0.05). In addition, the FMS of school-age children was moderately correlated with physical fitness levels (*r* = 0.358, *p* < 0.01). It can be seen that, except for emotional functioning, other variables in this study meet the preconditions of mediating effect analysis (34).

**Table 3:** Correlation analysis of variables.

Variable	1	2	3	4	5	6	7
1. FMS	1						
2. Physical functioning	0.244^**^	1					
3. Emotional functioning	−0.029	0.434^**^	1				
4. Social functioning	0.289^**^	0.396^**^	0.352^**^	1			
5. School functioning	0.301^**^	0.374^**^	0.429^**^	0.434^**^	1		
6. HRQoL	0.249^**^	0.717^**^	0.786^**^	0.719^**^	0.743^**^	1	
7. Physical fitness level	0.358^**^	0.232^**^	0.047	0.221^**^	0.263^**^	0.242^**^	1

*p < 0.05; **p < 0.01.

### 3.3. The mediating role of physical fitness level in fundamental movement skills and health-related quality of life

#### 3.3.1. Regression analysis of intermediary effect test

Table 4 shows the regression analysis of FMS, physical fitness levels, and HRQoL. The regression analysis results of controlling gender, age, and BMI – z scores showed that FMS significantly predicted the physical functioning (*β* = 0.319, *p* < 0.01), social functioning (*β* = 0.425, *p* < 0.01), and school functioning (*β* = 0.333, *p* < 0.01) of school-age children. When physical fitness levels enter the regression equation, the absolute value of the FMS regression coefficient decreases, but it can still significantly predict the physical functioning and school functioning of school-age children. It shows that physical fitness levels partially mediate between FMS, physical, and school functioning. However, when the physical fitness levels enter the regression equation, the physical fitness levels (*β* = 0.117, *p* > 0.05) are insignificant, indicating no intermediary role between FMS and social functioning.

**Table 4:** Regression analysis of FMS, physical fitness levels and HRQoL.

Variable			Gender	Age	BMI-z scores	FMS	Physical fitness level	*R*	*R* ^2^	*F*
Physical functioning	Model 1	*B*	−2.587	−1.104	−0.310	0.406		0.297	0.088	7.210
		*β*	−0.126	−0.125	−0.035	0.319				
		*t*	−2.153^*^	−1.374	−0.621	3.461^**^				
	Model 2	*B*	−3.387	−1.234	−0.214	0.317	0.260	0.353	0.125	8.487
		*β*	−0.166	−0.140	−0.024	0.249	0.211			
		*t*	−2.820^**^	−1.564	−0.437	2.691^**^	3.534^**^			
Social functioning	Model 1	*B*	2.392	−1.545	−1.345	0.591		0.351	0.123	10.478
		*β*	0.107	−0.160	−0.138	0.425				
		*t*	1.855	−1.793	−2.511^*^	4.697^**^				
	Model 2	*B*	1.907	−1.624	−1.287	0.537	0.157	0.366	0.134	9.240
		*β*	0.085	−0.168	−0.132	0.386	0.117			
		*t*	1.495	−1.892	−2.411^*^	4.190^**^	1.970			
School functioning	Model 1	*B*	4.025	−0.057	−0.389	0.461		0.354	0.125	10.698
		*β*	0.181	−0.006	−0.040	0.333				
		*t*	3.146^**^	−0.067	−0.732	3.691^**^				
	Model 2	*B*	3.440	−0.153	−0.319	0.396	0.190	0.377	0.142	9.849
		*β*	0.155	−0.016	−0.033	0.286	0.142			
		*t*	2.662^**^	−0.180	−0.604	3.122^**^	2.402^*^			

*p < 0.05; **p < 0.01.

#### 3.3.2. Bootstrap analysis of intermediary effect test

Table 5 is the Bootstrap analysis of physical fitness levels’ mediation effect test. The results showed that the 95%CI of physical fitness levels in school-age children’s FMS was [0.015, 0.195] and [0.007,0.150], respectively, excluding 0, which indicated the mediating effect of physical fitness levels between FMS and physical functioning and school functioning was significant. The value of the intermediary effect is 0.089 and 0.065, and the amount of intermediary effect is 21.89 and 14.11%, respectively.

**Table 5:** Bootstrap analysis of intermediary effect test of physical fitness levels.

Impact path	Standardization effect value	The ratio of total effect/%	Boot standard error	95% confidence interval	
				Lower limit	Upper limit
Indirect effectDirect effectTotal effectFMS…physical fitness levels…physical functioning	0.0890.3170.4060.089	21.89%78.11%21.89%	0.0460.1180.1170.046	0.0150.0850.1750.015	0.1950.5490.6360.195
Indirect effectDirect effectTotal effectFMS…physical fitness levels…School functioning	0.0650.3960.4610.065	14.11%85.89%14.11%	0.0370.1270.1250.037	0.0070.1460.2150.007	0.1500.6450.7060.150

## 4. Discussion

The main purpose of this study is to analyze the relationship between FMS, physical fitness levels, and HRQoL in school-age children; To explore the mediating role of physical fitness levels between FMS and HRQoL in school-age children. The results show that children with higher scores in FMS and physical fitness levels have better HRQoL than their peers with lower scores, mainly manifested in the overall score, physical functioning, social functioning, and school functioning of HRQoL. In addition, our research shows that physical fitness levels mediate the relationship between children’s FMS, physical functioning, and school functioning. Although the correlation of our report is small, it is consistent with the typical relationship between FMS and other outcome variables reported in previous studies (16). In order to evaluate the relationship between children’s FMS and HRQOL more accurately, it may be necessary to explore the factors influencing the relationship and reduce the influence of confounding factors on its correlation.

### 4.1. Influence of fundamental movement skills on health-related quality of life

School-age children’s FMS is positively correlated with physical functioning, social functioning, school functioning, and total score of HRQoL, which verifies hypothesis 1. This finding is consistent with other research results (16). FMS is a critical factor in school-age children’s ability to play and interact with others, and it is crucial for their future social interaction among peers (35). Children with poor FMS levels are more likely to withdraw from sports activities, increasing the possibility of relatively poor health (36). Low FMS levels make school-age children fatigued easily, thus restricting playground play, after-school activities, and other sports. In contrast, school-age children with high FMS levels are more capable of engaging in various activities or tasks and are more likely to keep healthy and contribute to school-age children’s social interaction because of more practice opportunities (22, 36). Longitudinal research shows that FMS of school-age children at 6–7 years old will affect their social interaction with peers at 9–10 (37). In addition, FMS level is related to better cognitive ability and can promote school-age children’s intelligence quotient (IQ), attention, inhibition and control, memory, and academic performance tasks (38). Based on previous studies, we can think that compared with school-age children with low FMS levels, school-age children with high FMS levels may participate in organized or accessible physical activities, concentrate more easily on learning tasks, and experience less social problems (15, 38), which makes them feel better in sports, physical and motor skills. Therefore, the FMS development of school-age children is related to their better physical functioning, social functioning, school functioning, and HRQoL.

School-age children with negative emotions are more likely to have internalization and externalization problems and experience peer rejection, thus affecting their overall mental health and well-being (39, 40). Studies have shown that the development level of FMS may positively impact the emotional functioning of school-age children, such as self-confidence, self-esteem, depression, and anxiety (41). However, the results of this study show that FMS has no significant correlation with emotional functioning, which may be due to other factors such as family environment and peer interaction. For example, the experience of disharmony in the family relationship will put school-age children at risk of developing emotional functioning, and they are prone to depression or anxiety. At the same time, peer rejection and social marginalization will also adversely affect school-age children’s emotions (42, 43). Therefore, to analyze the relationship between FMS and emotional functioning, it is necessary to consider the interference of other confounding factors comprehensively.

### 4.2. Influence of physical fitness level on health-related quality of life

A significant positive correlation exists between children’s physical fitness levels and HRQoL, which verifies hypothesis 2. This finding is consistent with other research results (44, 45). The physical fitness measurement items of school-age children in this study include BMI, vital capacity, 50-meter running, sitting body flexion, one-minute jump rope, one-minute sit-ups, etc. Other countries are mainly muscle strength and endurance (sit-ups, grip strength, standing long jump), cardiopulmonary fitness (20-meter turn-back run), etc. (46, 47). Although the test items of physical fitness levels in China differ from those in other countries, physical fitness levels positively impact the HRQoL of school-age children. Specifically, physical fitness levels are positively correlated with the physical, social, and school functioning of HRQoL. School-age children with high physical fitness usually show higher physical activity levels, academic performance, and behavior adjustment ability in later life, thus improving HRQoL (48). However, there is no significant correlation between physical fitness levels and emotional functioning. Basterfield et al. (49) found a slight but significant positive correlation between physical fitness levels and emotional functioning. In comparison, Franquelo et al. (44) found a correlation between girls’ physical fitness levels and emotional functioning. However, there was no significant correlation between them in boys. Therefore, there is no consistent conclusion about the relationship between physical fitness levels and emotional functioning, and more research is needed to explore the relationship between them further.

### 4.3. The mediating role of physical fitness level in fundamental movement skills and health-related quality of life

This study found that physical fitness levels partially mediate between FMS, physical functioning, and school functioning of school-age children; that is, FMS can indirectly affect the physical functioning and school functioning of HRQoL of school-age children through physical fitness levels, which verifies hypothesis 3. Redondo-Tebar et al.’ s (16) research show that physical fitness levels are intermediary in male students’ FMS, physical, and school functioning. This study further verified the research results of Redondo-Tebar et al. (16). Many studies have shown that the FMS level promotes school-age children to participate in diversified and high-intensity sports activities, which provides them with various sports experiences. Compared with their peers, school-age children’s efficient exercise mode means they have less fatigue and lower energy consumption, which promotes the development of daily physical functioning of school-age children, such as walking/running ability, lifting weights, and other physical functioning (11). In addition, FMS can promote the school functioning of school-age children through Physical fitness levels. FMS improves school-age children’s physical fitness and social and cognitive abilities (50). Compared with school-age children with low FMS levels, children with high motor skills have good physical fitness. They can concentrate on school activities for a long time, so they have less school functioning, such as absence from school and difficulty completing their studies (51). Good FMS can positively impact children’s physical and school functioning by improving physical fitness levels. Therefore, improving FMS and physical fitness levels may be a practical strategy to improve children’s HRQoL. Family and education departments should promote effective intervention measures and integrate them into various activities inside and outside children’s schools to improve FMS and physical fitness levels while maximizing school-age children’s HRQoL.

## 5. Strengths and limitations

This study investigates the relationship between FMS, physical fitness levels, and HRQoL of typical school-age children. The variables (FMS, physical fitness levels, and HRQoL) are measured with high standardization. However, some limitations must be emphasized, which need to be improved in follow-up research. First, this is a cross-sectional design, so the ambiguity in time hinders the causal inference. In future research, a longitudinal investigation of the same sample can be considered to verify the prediction effect of FMS and physical fitness levels on HRQoL. Second, other factors not evaluated in this study may affect the results (such as eating habits, family environment, and social and economic status). In future research, we should explore the factors that influence the relationship between FMS, physical fitness levels, and HRQoL of school-age children to improve the accuracy of the research. Thirdly, because of the participants’ age, we used the parents’ agent to report the PedsQL™4.0 questionnaire instead of the self-agent report. Therefore, future research should consider using practical self-reporting tools in younger children.

## 6. Conclusion

This study investigated 6-10-year-old school-age children and found that FMS and physical fitness levels can positively predict HRQoL and physical, social, and school functioning. Physical fitness levels play a partial intermediary role between the physical and school functioning dimensions of FMS and HRQoL.The development of skilled FMS is vital in promoting school-age children’s health and improving HRQoL. Therefore, we suggest that the family and education departments promote effective intervention measures to improve FMS and physical fitness levels while maximizing school-age children’s HRQoL. The findings of this study are of great significance to improving children’s overall health and life satisfaction in the future.

## Data availability statement

The datasets presented in this study can be found in online repositories. The names of the repository/repositories and accession number(s) can be found in the article/supplementary material.

## Ethics statement

The studies involving human participants were reviewed and approved by the Zhejiang Normal University Ethics Committee. The participants legal guardian/next of kin provided their written informed consent to participate in this study. In the data collection process, the children were instructed by school teachers to understand the research purpose, social value, and benefits. School-age children, parents, and teachers are told the scope of information to be collected, the possible risks involved in privacy, and the measures to be taken. Written informed consent was obtained from the individuals' legal guardians/next of kin for the publication of any potentially identifiable images or data included in this article.

## Author contributions

SX wrote the first draft of the paper. YY, RS, and LF performed data calculation and analysis. WS and YZ critically revised the work. All authors commented on previous versions of the paper, read and approved the final version, and contributed to the research conception and design.

## Conflict of interest

The authors declare that the research was conducted in the absence of any commercial or financial relationships that could be construed as a potential conflict of interest.

## Publisher’s note

All claims expressed in this article are solely those of the authors and do not necessarily represent those of their affiliated organizations, or those of the publisher, the editors and the reviewers. Any product that may be evaluated in this article, or claim that may be made by its manufacturer, is not guaranteed or endorsed by the publisher.
